# Thermal Profiles of Chainsaw Hollows and Natural Hollows during Extreme Heat Events

**DOI:** 10.3390/biology12030361

**Published:** 2023-02-24

**Authors:** Michael N. Callan, Dan Krix, Christopher M. McLean, Brad R. Murray, Jonathan K. Webb

**Affiliations:** 1 Habitat Innovation and Management, 30 Lorimer Street, Llanarth, NSW 2795, Australia; 2 School of Life Sciences, University of Technology Sydney, Broadway, NSW 2007, Australia; 3 Specialist Research Services, Gosford, NSW 2250, Australia

**Keywords:** heatwave, thermal extremes, artificial hollow, marsupial, upper critical temperature, heat stress

## Abstract

**Simple Summary:**

Loss of large hollow-bearing trees is a major threat to the persistence of wildlife in agricultural landscapes. To combat this problem, local governments, volunteer environmental organizations, and conservation groups increasingly carve artificial hollows (“chainsaw hollows”) in dead trees, yet little is known about their thermal profiles. We measured the thermal profiles of 13 natural and 45 artificial hollows in the central west of NSW, Australia, over the course of 2 summers. Maximum temperatures and daily temperature ranges within natural hollows and artificial hollows were similar in 2017–2018. During the January 2019 heatwave, temperatures inside chainsaw hollows in dead trees exceeded 35 °C for 6.2 consecutive days. Artificial hollows in dead trees provided little buffering from thermal extremes; when air temperatures peaked at 44.6 °C, hollow temperatures were only 2.4 °C cooler on average than ambient (range: 5.5 °C cooler to 1.0 °C hotter than ambient). Artificial hollows created in dead trees may therefore not provide suitable thermal conditions for hollow-dependent wildlife during hot summers. Thus, retention of large live trees, coupled with revegetation, is crucial for conserving hollow-dependent fauna in agricultural landscapes.

**Abstract:**

Loss of hollow-bearing trees threatens many hollow-dependent wildlife. To mitigate this process, artificial chainsaw-carved hollows (CHs) are often created in dead trees, yet little is known about their thermal profiles. We measured temperatures inside 13 natural hollows (8 live and 5 dead trees) and 45 CHs (5 live and 40 dead trees) in the central west of NSW, Australia, over the course of 2 summers. Maximum temperatures and daily temperature ranges within natural hollows and artificial hollows were similar in 2017–2018. Hollow temperatures were lower in thicker-walled hollows than in thinner-walled hollows. During the January 2019 heatwave, temperatures inside CHs in dead trees exceeded 4–35 °C higher than the upper limit of the thermal neutral zone of sugar gliders—for 6.2 consecutive days (range 0–9 days). CHs in dead trees provided little buffering from thermal extremes; when air temperatures peaked at 44.6 °C, CHs in dead trees were on average 2.4 °C cooler than ambient (range: 5.5 °C cooler to 1.0 °C hotter than ambient). These results show that CHs created in dead trees may not provide suitable thermal conditions for hollow-dependent marsupials during summer heatwaves. Retention of large live trees, coupled with revegetation, is crucial for conserving hollow-dependent fauna in agricultural landscapes.

## 1. Introduction

Australia has one of the world’s worst records for tree clearing. In the last 200 years, over 40% of Australia’s native forests have been cleared [1,2], leading to a countrywide decline in numbers of large hollow-bearing trees [3,4]. Because Australia has no vertebrates that actively excavate hollows in living trees, it can take decades for smaller hollows to form [5], and centuries for the creation of large hollows [6,7,8]. The loss of hollows can therefore have major effects on populations of hollow-dependent fauna [8,9,10]. In NSW, loss of tree hollows is listed as a key threatening process under the Threatened Species Conservation Act, and is considered a major contributor to the decline of endangered hollow-dependent wildlife [11].

In an effort to conserve hollow-dependent animals, councils, community groups, and conservation organizations have installed wooden nest boxes in conjunction with revegetation programs. However, nest boxes suffer from several problems, such as non-usage by target species, occupancy by invasive species, and high costs associated with ongoing maintenance [12,13,14]. In addition, wooden nest boxes lack the insulative properties of hollows in live trees, and so temperatures inside nest boxes typically fluctuate more than those inside natural hollows [15,16,17]. This can be particularly problematic during heatwaves, when temperatures inside nest boxes can often exceed 40 °C [18], which can lead to heat stress or the death of animals using the hollows [19]. To mitigate these problems, chainsaw-carved hollows (henceforth, CHs) in which hollows are carved directly into live or dead trees [20,21], have increasingly become an alternative to nest boxes in Australia [14,22,23].

While several studies have shown that a diversity of fauna will use CHs [22,23,24,25], less is known about their thermal properties. Several recent studies carried out in southern Australia found that temperatures inside CHs and natural hollows in live trees were very similar, with both structures providing temperatures that were warmer than ambient at night, and cooler than ambient during the day [15,26]. Importantly, a recent study by Griffiths et al. (2022), found that even when ambient temperatures reached 46.5 °C, mean temperatures inside chainsaw-carved hollows in live trees were 30.2 °C, demonstrating that such structures can provide thermal refuges to wildlife during extreme heat events [26].

Although the results of Griffiths et al. (2022) are encouraging, they were based on temperatures recorded inside CHs created in 8 mature live trees at heights of 1.5 to 2.5 m above ground [26]. However, in many rural and agricultural landscapes, large live trees suitable for creation of hollows are scarce [27] and so CHs are often created in standing dead trees. Because dead trees lack the shade provided by a canopy, and the thermal insulation provided by the flow of water within the sapwood of live trees [28], it is likely that CHs carved into dead trees are hotter than has been reported for CHs carved into live trees. As far as we know, there are no published studies on the thermal properties of CHs carved into dead trees [26], so we do not know if such structures provide thermally suitable diurnal refuges for nocturnal marsupials during summer, particularly during heatwaves. To address this knowledge gap, we measured the thermal profiles of CHs carved into dead trees in the central west of NSW, a region which experiences hot dry summers. We also measured the thermal profiles within natural hollows in live and dead trees, and within CHs carved into live trees. Specifically, we investigated five questions: (1) Are the thermal profiles of CHs carved into dead trees similar to those within natural hollows? (2) Is the daily temperature range inside CHs and natural hollows similar? (3) Are the maximum daily temperatures of CHs similar to those of natural hollows? (4) Do temperatures in artificial and natural hollows exceed the upper critical temperatures of arboreal marsupials, and if so, for how long each day? (5) Are CHs in dead trees buffered from high ambient temperatures, as is the case for CHs carved into live trees [26]?

## 2. Materials and Methods

### 2.1. Study Area and Hollow Creation

We worked on a revegetation and hollow creation project led by the Central Tablelands Local Land Services and the Environment & Waterways Alliance in which artificial hollows were created in standing trees to provide habitats for hollow-dependent wildlife, including the threatened superb parrot *Polytelis swainsonii*. Hollows were created in trees at 11 sites spread across the Bathurst, Blayney, Cabonne, Cowra, and Orange Local Government Areas in the central west of NSW, a region that is dominated by dryland agriculture and plantations [24]. Native vegetation in the region comprises grassy woodlands, dry sclerophyll forest, and floodplain transition woodlands [29]. A total of 187 artificial hollows were installed by qualified arborists between 2017 and 2018 in standing dead trees and live trees in which the ratio of solid wall thickness (t) over trunk radius was >0.3 [24]. This criterion was used because it provides a reasonable estimate of the likelihood of tree failure, specifically reducing the structural integrity of the tree leading to collapse [30,31]. Other criteria which influenced tree selection included the health of the tree, the location of the tree, the potential risk posed to the public should the tree fail, and the safety of arborists and tree climbers carrying out subsequent works on the tree. Live trees included *Eucalyptus albens*, *E. blakelyi*, *E. bridgesiana*, *E. melliodora*, and *Eucalyptus viminalis*. Hollows were created based on the methodology developed by Gano and Mosher [21] following the steps outlined in 2010 by Kenyon and Kenyon [32], whereby a chainsaw was used to remove a timber slab or faceplate from the tree before a cavity was carved into the tree. The faceplate was then screwed into place to enclose the cavity, and an entrance hole was then cut into the faceplate to allow fauna to access the hollow [14,24]. Internal hollow dimensions ranged between 30–86 cm high and 12–40 cm wide, and heights varied from 7 to 20 m. Entrance holes ranged between 45–100 mm, relative to the size of the created cavity. The mean diameter at breast height of trees containing natural hollows was 99 cm (range 55–240 cm) and for CHs it was 95 cm (range 50–200 cm), while depth of natural hollows ranged between 30 cm to over 100 cm (for safety and logistical reasons, the depth of 6 natural hollows could not be accurately measured).

### 2.2. Monitoring Cavity Temperatures

To measure internal hollow temperatures, we used miniature data loggers (Thermochron i-button, DS1922L, range −40–85 °C, accuracy ± 0.5 °C, Analog Devices, Wilmington, USA) affixed to 19 mm diameter dowel sticks. The data loggers were programmed to record temperatures every hour and were glued to the ends of the dowels. For each hollow, an experienced tree climber ascended the tree and drilled a 20 mm diameter hole approximately 150 mm below the hollow entrance before inserting the dowel stick into the hollow such that it protruded approximately 10–20 mm from the inner wall. The dowel was then sealed in place with window putty. We sampled temperatures inside a total of 71 hollows, but 6 loggers were lost, and 7 failed to record data or had erroneous readings due to battery failure, so the final sample size was 58 hollows. Hollows were accessible to wildlife throughout the duration of the study, with fauna presence acknowledged as likely to influence cavity temperatures, but that is beyond the scope of this work to address.

Air temperature data recorded from the weather station closest to each hollow location were sourced from the Bureau of Meteorology (BOM) to provide ambient temperature data for each of the hollows for which we had temperature data. Data from four weather stations (Bathurst, Cowra, Orange and Wellington) were downloaded from the BOM climate website (http://www.bom.gov.au/climate/data/, accessed 22 July 2022). Ambient temperatures varied throughout the region during the two summers. In the summer of 2017–2018, mean daily maxima ranged from 19.2 to 40.1 °C, while mean daily minima ranged from 8.0 to 23.0 °C. Across the 4 weather stations, there were, on average, 2.3 days where daily maxima exceeded 40.0 °C (range 0–5 d), and 20.8 days where daily maxima exceeded 35 °C (range 0–38 d). The summer of 2018–2019 was considerably hotter, with mean daily maxima ranging from 20.8 to 41.0 °C and mean daily minima ranging from 7.4 to 24.6 °C. Across the four weather stations, there were, on average, 5.3 days where daily maxima exceeded 40.0 °C (range 0–12 d) and 20.8 days where daily maxima exceeded 35 °C (range 5–52 d). An extended heatwave, defined as 3 or more days where temperatures exceed the calendar day 90th percentile [33] occurred in the region from 12–20 January 2019, when daily maxima at Wellington ranged from 39.3 °C to 44.6 °C, and daily minima ranged from 20.0 to 27.0 °C.

### 2.3. Statistical Analyses

The dataset consisted of 45 trees with artificial hollows and 13 with natural hollows, distributed among 10 sites, measured across 2 summers (2017–2018, and 2018–2019). The majority of artificial hollows were cut in dead trees (40 of 45), with natural hollows in 5 dead trees, and 8 in live trees. Approximately a quarter of the artificial hollows were located in a branch (12 hollows), and 33 in a trunk. Five of the natural hollows occurred in a branch and eight in a trunk. We obtained temperature data from 26 artificial hollows, and 10 natural hollows in the 2017-2018 summer, and 30 artificial hollows and 4 natural hollows in the 2018–2019 summer. A total of 12 hollows monitored in the first summer (11 CHs, 1 natural) were also monitored in the second summer. For all hollows, we obtained 90 days of continuous temperature data over the summer. Raw data used in statistical analyses can be found in S1 of the Supplementary Materials.

From the collected temperature data, the daily maximum temperature, the daily temperature range, and the daily count of hours above 35 °C were calculated for each hollow. The number of hours above 35 °C is biologically relevant because nocturnal mammals can experience heat stress when ambient temperatures exceed their upper critical temperatures [34,35,36]. The upper critical temperatures of arboreal mammals in our study area range from 31.0 °C in *Petaurus notatus* to 35.1 °C in *Acrobates pygmaeus* [35,37]. Using the daily records as replicates, linear mixed models (LMMs) were employed to model daily temperature range and maximum temperature, and a generalized linear mixed model (GLMM) with a gamma error distribution and log link was employed to model the counts of hours above 35 °C. To test the effects of wall thickness (sqrt transformed continuous factor) and tree status (categorical fixed factor; tree dead or alive), models were fitted with these terms, including a random effect of tree nested within site and a fixed effect for hollow type (two level categorical; AH or CH). A further interaction term of hollow type x tree status was included to test for differences among live CH and AHs, or dead CHs and AHs. These models used the first season’s data (summer of 2017–2018) only, as we had insufficient replicates in the second year. A further set of models using both summers’ data were then fitted, to quantify the difference between the summers. These included a term for the summer (two level categorical; summer of 2017–2018 and 2018–2019), hollow type, a hollow type × summer interaction (to test if effects were consistent between summers for the hollow types), and wall thickness, with random terms for tree, nested within site, and nested within summer. The structure of the random terms allowed us to control for trees and sites measured across summers, or only a single summer. All analyses were conducted in R 4.1.2 [38], using the packages beeswarm [39], car [40], emmeans [41], and lme4 [42]. Results from emmeans [41] were used to present estimated marginal means (i.e., the effect of interest averaged across the levels of all other categorical factors in the models) of model effects graphically for all categorical variables.

## 3. Results

### 3.1. Daily Thermal Profiles of CHs and Natural Hollows

**Figure 1:**
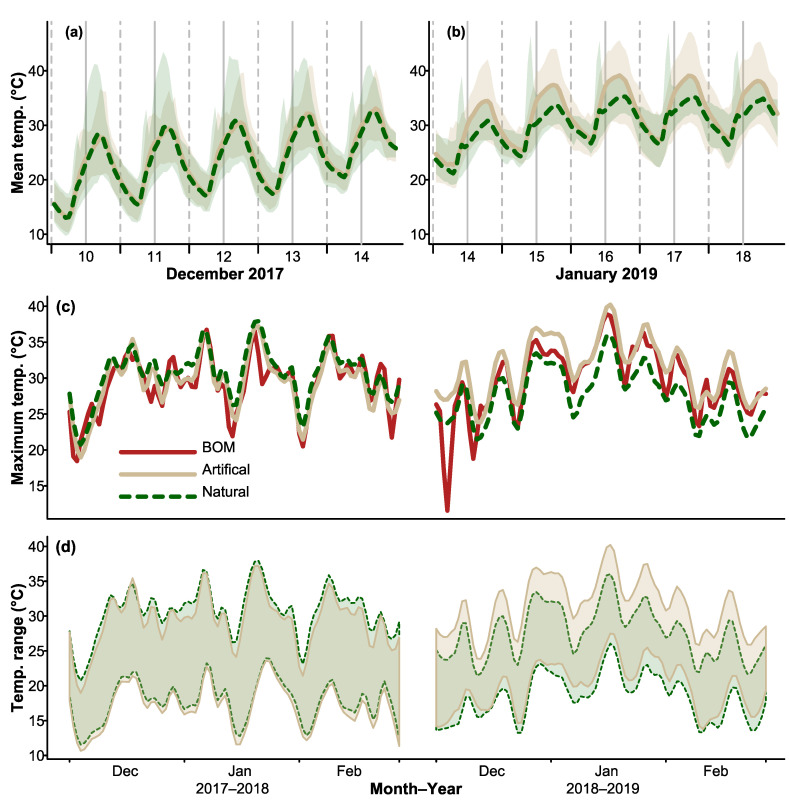
Mean trends in (**a**,**b**) mean temperature, (**c**) maximum temperature, and (**d**) temperature range. Trends were taken from an average across stations (loess smoothed for clarity). A color key is shown in middle left panel in (**c**). The Bureau of Meteorology air temperature data (BOM) shown in (**c**) represents a weighted average of maximum daily air temperatures recorded from the nearest stations to the hollow locations.

During the summer, thermal profiles of natural hollows and CHs displayed similar daily fluctuations, with temperatures in both structures reaching a peak around 4 p.m. each day and decreasing to a minimum around 6 a.m. (Figure 1a). During the heatwave in January 2019, similar patterns were observed, but mean and maximum temperatures were higher than those recorded during the hottest days in December 2017 (Figure 1b). Plots of maximum daily temperatures recorded inside hollows and maximum daily air temperatures showed that thermal maxima of hollows tracked maximum daily air temperatures (Figure 1c). Temperature ranges recorded inside hollows showed a very similar pattern, indicating that hollow temperatures were influenced by ambient conditions (Figure 1d).

### 3.2. Daily Temperature Ranges in CHs and Natural Hollows

**Figure 2:**
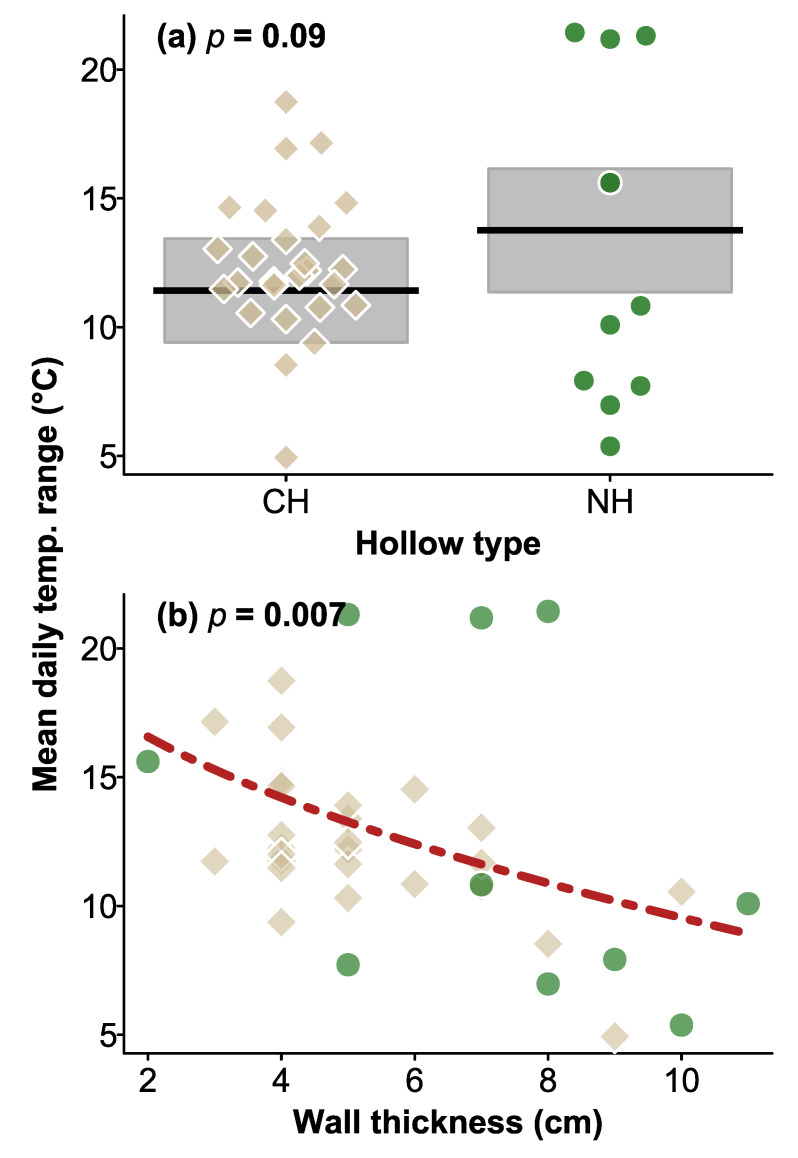
Plot of daily mean temperature range for (**a**) CHs and natural hollows and the relationship with wall thickness. In (**a**) shaded area are the 95% CI of the mean (black lines), the broken red line in (**b**) shows the relationship between hollow temperature range and hollow wall thickness, averaged across the categorical factors in the analysis. Brown squares and green circles in (**b**) indicate CHs and natural hollows, respectively, as shown in (**a**), indicating mean values for hollows in the 2018–2019 summer. *p* values for the effect of hollow type (**a**) and wall thickness (**b**) are shown at the top of the plots.

Daily temperature ranges within hollows varied from 1.5–33.0 °C in natural hollows and 2.0–33.5 °C in CHs. Overall, temperature ranges were very similar within natural and CHs, with no significant difference between hollow types (χ^2^ = 2.950, df = 1, *p* = 0.09; Figure 2), tree status (χ^2^ = 1.796, df = 1, *p* = 0.2), and no significant interaction between hollow type and tree status (χ^2^ = 0.509, df = 1, *p* = 0.5). Natural hollows had slightly thicker walls than CHs (means of 7.1 versus 5.3 cm, respectively, F_1.56_ = 9.86, *p* = 0.003). Overall, thicker-walled hollows had a smaller temperature range (χ^2^ = 7.152, df = 1, *p* = 0.007) than thinner-walled hollows (Figure 2).

**Figure 3:**
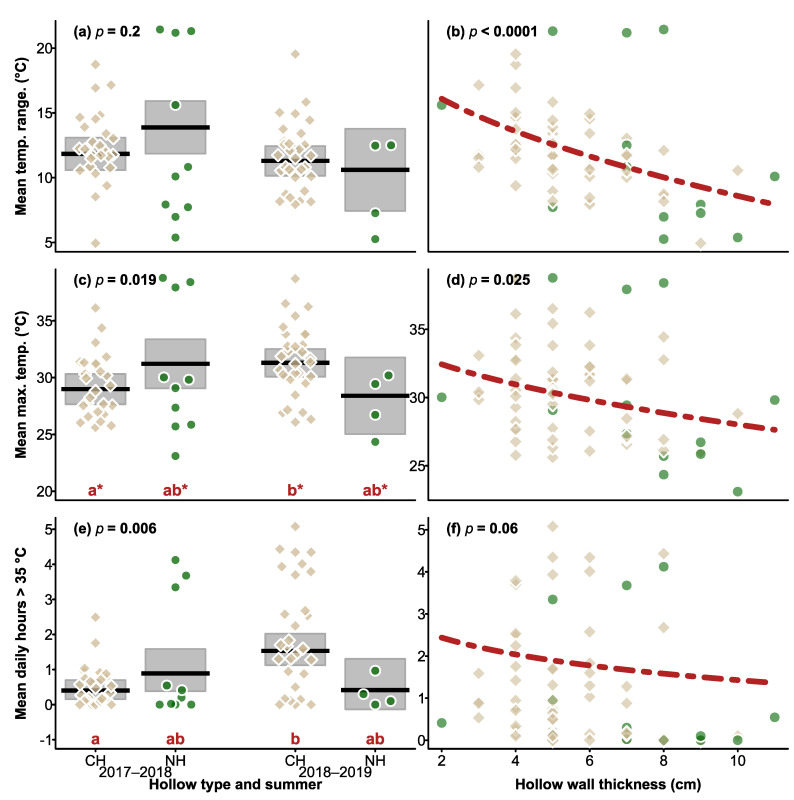
Plots of daily mean temperature range (**a**,**b**), mean maximum temperature (**c**,**d**) and hours over 35 °C (**e**,**f**), over the two summers by hollow type (CH = chainsaw-carved hollow, NH = natural hollow) and summer (left column), and the responses relationship with wall thickness (right column). At the top of the plots showing hollow types × summer the *p* value of hollow type × summer interaction term is shown, and the significance of the effect of wall thickness is shown in the right column of the plots. For (**c**,**e**), the pattern of differences among the groups is indicated by the red letters on the x-axis, with the asterisk indicating statistically significant differences. In (**c**), no significant patterns emerged after correction for multiple comparisons, so results presented here are shown without correction to indicate which groups created the significant interaction effect. In the left column, means are indicated by black lines, shaded areas the 95% CI, and in the right column the broken red line indicates the relationship between hollow wall thickness, averaged across the categorical factors in the analysis, and a given response. Brown squares and green circles indicate mean values for CHs and NHs respectively within (left column) or across (right column) summers.

When we compared the two summers, no significant difference was found between hollow type (χ^2^ = 1.201, df = 1, *p* = 0.3), summer (χ^2^ = 1.669, df = 1, *p* = 0.2), or the summer × hollow type interaction (χ^2^ = 1.669, df = 1, *p* = 0.2; Figure 3). As before, thicker-walled hollows showed a smaller variation in daily temperatures than thinner-walled hollows (χ^2^ = 16.424, DF = 1, *p* < 0.0001; Figure 3).

### 3.3. Maximum Daily Temperatures in CHs and Natural Hollows

**Figure 4:**
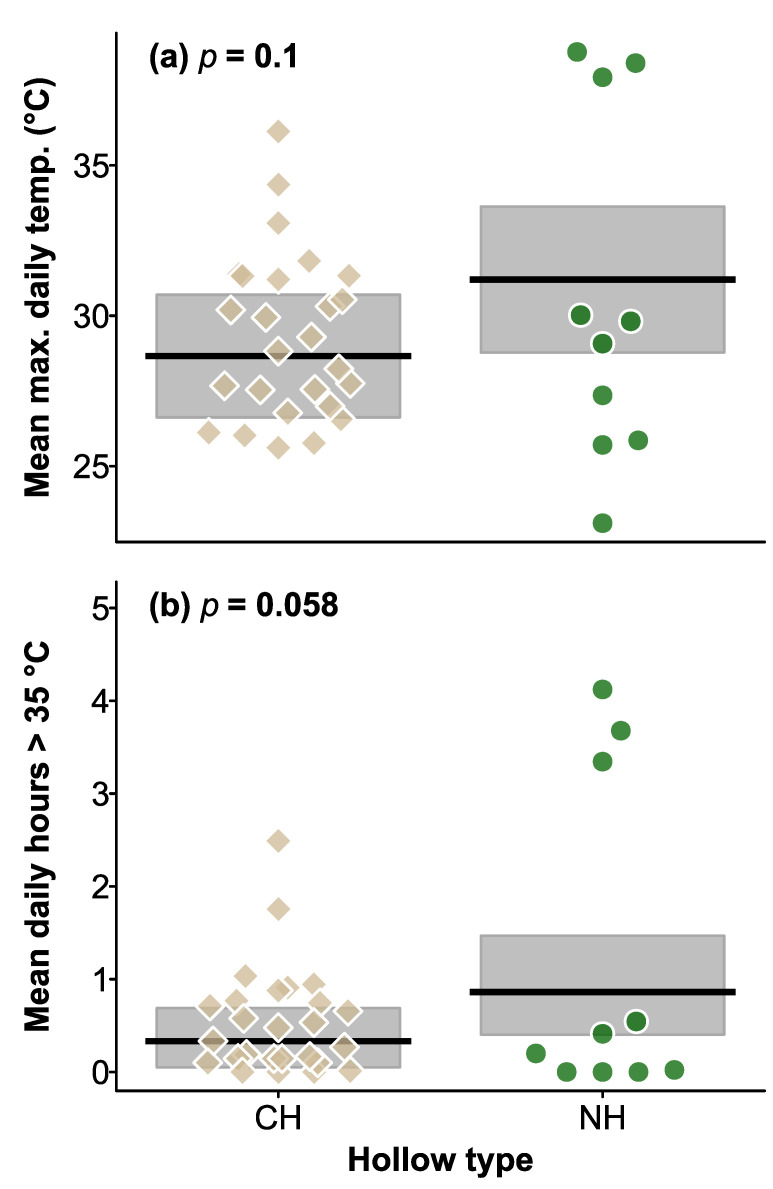
Plot of daily mean maximum temperature (**a**) and mean daily hours over 35 °C (**b**) in CHs and NHs. Shaded area are the 95% CI of the mean (black lines). *p* values for the effect of hollow type are shown at the top of the plots. Brown squares and green circles indicate mean values for CHs and NHs respectively in the 2018–2019 summer.

Maximum daily temperatures recorded inside CHs and natural hollows ranged from 13.5 to 50.5 °C, and 17.6 to 52.5 °C, respectively. In the first year, maximum temperatures did not differ between hollow types (χ^2^ = 2.660, df = 1, *p* = 0.1; Figure 4), tree status (live or dead, χ^2^ = 0.330, df = 1, *p* = 0.6), wall thickness (χ^2^ = 3.020, df = 1, *p* = 0.08), and the interaction between hollow type and tree status was also not significant (χ^2^ = 0.006, df = 1, *p* = 0.9).

When we compared maximum daily temperatures across the two summers, a significant hollow type × summer effect emerged (χ^2^ = 5.476, df = 1, *p* = 0.019; Figure 3), however the effect was small, and after adjustment for pairwise testing, the pattern was not significant. Without adjustment, maximum temperatures of CHs differed between summers (Figure 4), but there were no differences between hollow types within each summer. Overall, thicker walled hollows had lower maximum temperatures (χ^2^ = 5.043, df = 1, *p* = 0.025; Figure 4), but there was no difference between hollow types (χ^2^ = 0.235, df = 1, *p* = 0.6), or between summers (χ^2^ = 3.045, df = 1, *p* = 0.08).

### 3.4. Number of Hours > 35 °C in Natural Hollows and CHs in Summer, and during the 2019 Heatwave

In the first year, the number of hours > 35 °C was not influenced by hollow type (χ^2^ = 3.582, df = 1, *p* = 0.058; Figure 4), tree status (χ^2^ = 1.095, df = 1, *p* = 0.3), wall thickness (χ^2^ = 2.201, df = 1, *p* = 0.1), or interaction between hollow type and tree status (χ^2^ = 0.061, df = 1, *p* = 0.8).

When we compared the two years, there was no effect of hollow type (χ^2^ = 0.003, df = 1, *p* = 1), but the hollow type x summer interaction was significant (χ^2^ = 7.666, df = 1, *p* = 0.006), and was driven by CHs in 2018–2019 recording more hours > 35 °C (mean = 2.0 h) compared to CHs in 2018–2018 (mean = 0.54 h). No significant effect of wall thickness on the number of hours > 35 °C was detected (χ^2^ = 3.496, df = 1, *p* = 0.06; Figure 3).

**Figure 5:**
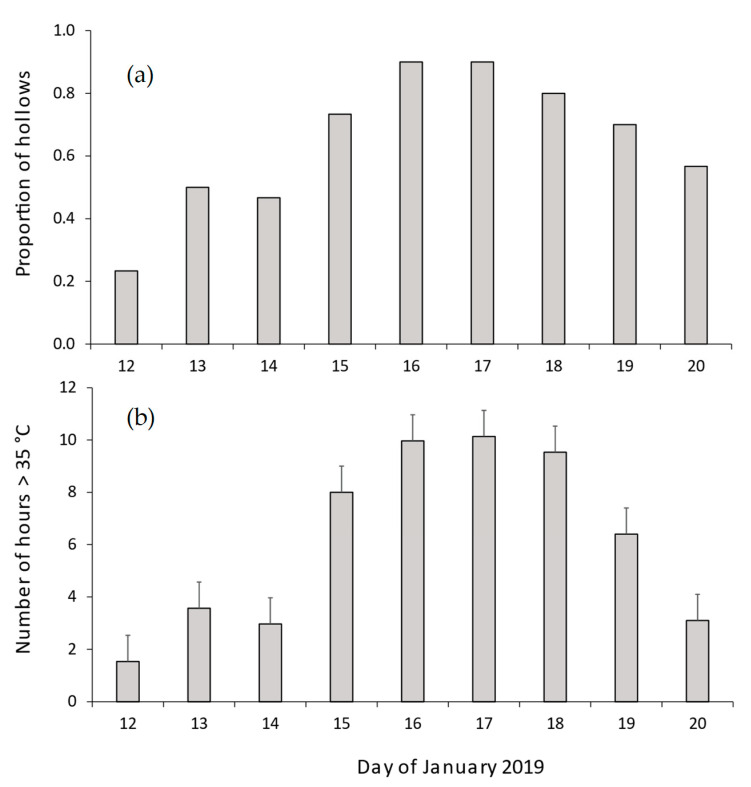
Thermal characteristics of CHs during the January 2019 heatwave. The figure shows (**a**) proportion of chainsaw-carved hollows where temperatures exceeded 35 °C for more than two hours per day and (**b**) the mean number of hours per day in which hollow temperatures exceeded 35 °C. Error bars in panel (**b**) denote standard errors.

We also examined the thermal profiles of 30 CHs during the prolonged heatwave (9–20 January 2019). In all but 3 CHs, temperatures exceeded 35 °C for 6.2 consecutive days (range 0–9 d). The proportion of sampled hollows where temperatures exceeded 35 °C for more than 2 h each day ranged from 0.23–0.90 (Figure 5a). The mean number of hours per day during which hollow temperatures exceeded 35 °C was 6.1 h (range 1.5–10.1 h per day, Figure 5b).

### 3.5. Thermal Buffering within Hollows

**Figure 6:**
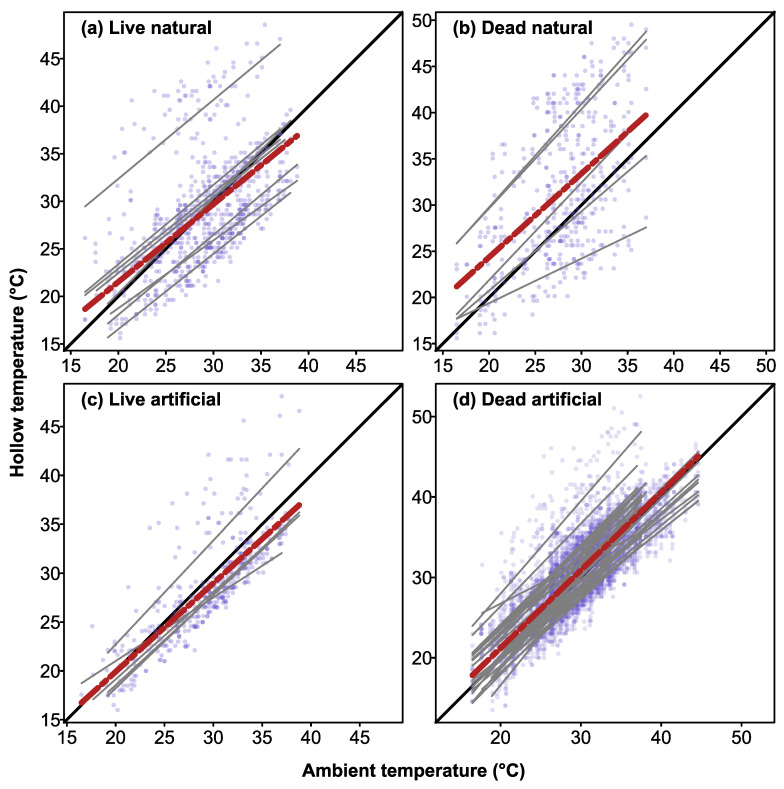
Linear regressions of relationship between maximum daily air temperature (recorded at the weather station nearest to the hollow) and maximum hollow temperature for natural hollows in live (**a**) and dead (**b**) trees, and artificial CHs in live (**c**) and dead (**d**) trees. Grey solid lines show linear fits for individual hollows, while black solid line indicates a 1:1 relationship between ambient temperature and hollow temperature. Lines below the 1:1 relationship indicate thermal buffering. Red dotted lines show lines of best fit for all hollows combined, while blue dots indicate data points for individual hollows.

There were significant positive correlations between maximum daily air temperatures recorded at the nearest weather station versus maximum temperatures recorded inside CHs in live trees (CHs: *r* = 0.81, *r*^2^ = 0.65, *p* = 0.001) and dead trees (*r* = 0. 79, *r*^2^ = 0.63, *p* = 0.001). For natural hollows, there were also positive correlations between maximum daily air temperatures and maximum hollow temperatures in live trees (*r* = 0.45, *r*^2^ = 0.21, *p* = 0.001) and dead trees (*r* = 0.47, *r*^2^ = 0.22, *p* = 0.001). All the relationships between hollow temperatures and maximum daily air temperatures differed from a slope of one (equality with ambient air temperature; all tests < 0.05). Inspection of plots of maximum daily air temperatures versus maximum hollow temperature showed that some natural hollows and CHs were buffered from high temperatures, whereas others were not (Figure 6).

## 4. Discussion

Chainsaw-carved hollows are increasingly being used in conservation programs, and a diversity of wildlife have been reported to use these structures [22,23,24,25]. However, little is known about the thermal profiles of CHs carved into dead trees, particularly during summer heatwaves [26]. We measured the thermal profiles of CHs and natural hollows in dead and live trees in the central west of NSW over two consecutive summers, which included one of the hottest summers on record. With respect to our original questions, we found that (1) the daily thermal profiles of CHs in dead trees were similar to those of natural hollows, (2) the daily temperature ranges of CHs and natural hollows were similar, and (3) the maximum daily temperatures of CHs in dead trees were similar to those of natural hollows (Figure 2 and Figure 3). Hence, the CHs in dead trees had similar thermal properties to those of the natural hollows that we sampled. While this result is reassuring, we also found that (4) CHs in dead trees were poorly buffered from high ambient temperatures, and (5) temperatures in CHs (and some natural hollows) exceeded the upper critical temperatures of marsupials for several hours per day. We discuss these findings below.

Despite having similar thermal profiles to natural hollows, the CHs carved into dead trees lacked the thermal buffering properties that have been reported for CHs carved into large live trees in southern Australia [26]. Maximum daily temperatures recorded inside CHs in dead trees were positively correlated with maximum daily air temperatures, and while some hollows were buffered against high ambient temperatures, others were not (Figure 6d). During the January 2019 heatwave, when air temperatures peaked at 44.6 °C, CHs in dead trees were, on average, 2.4 °C cooler than ambient (range: 5.5 °C cooler to 1.0 °C hotter than ambient). By contrast, a recent study conducted during the same period (January 2019), found that CHs in large live trees were very well insulated against high temperatures, with internal temperatures 16 °C lower than ambient (range 10–18.8 °C) on the hottest day when ambient temperature was 45 °C [26]. This difference likely reflects two factors. First, the mean wall thicknesses of the CHs we sampled was 5.3 cm, which was likely insufficient to insulate them from thermal extremes. In this respect, the CHs we sampled were very similar to the log hollows sampled by Griffiths et al. [15], which had mean wall thicknesses of 4.7 cm, and had thermal variation that was in between that of nest boxes and CHs carved into live trees. Second, the CHs we sampled lacked the thermal insulation provided by the flow of water within the sapwood of live trees [28]. As trees get larger in diameter at breast height, the area of sapwood increases [43], and thus, the cooling capacity provided by sapwood flow is likely greatest in large live trees. Sapwood temperatures also show vertical gradients in live trees, with lower temperatures in lower stems than in upper crowns [44,45], which would likely also contribute to thermal buffering within low hollows in live trees. Furthermore, most of the hollows that we monitored were located in dead trees, at heights between 7 to 20 m above ground, and so would have received high solar radiation loads [44]. By contrast, the CHs constructed in the previous study [26] were carved into large mature live trees at heights of 1.5 to 2.5 m, and so would likely have been shaded by the canopy.

As a consequence of their poor thermal buffering capacity (Figure 6), temperatures in CHs, and some natural hollows, exceeded 35.0 °C (4 °C higher than the thermal neutral zone of gliders) for several hours each day (Figure 4e and Figure 5). While many species of arboreal marsupials can tolerate brief exposures to ambient temperatures above their thermal neutral zone [46,47], one study reported the death of a sugar glider after just 2 h exposure to 38 °C [35]. Prolonged exposure to temperatures above the animals’ thermal neutral zone can result in significant increases in metabolism and water loss [16,34], increasing the risks of death from dehydration [48]. During the January 2019 heatwave (12–20 January), temperatures in 70% of hollows exceeded 35 °C for 6 h per day on 5 consecutive days (Figure 5) and animals using such hollows would therefore experience significant heat stress and water loss unless they sought shelter in cooler hollows [48]. While CHs and natural hollows in live trees could provide cooler microsites for wildlife during heatwaves [26,49], dead trees often account for a significant proportion of the available trees on farmland and regrowth forests throughout Australia [27]. Hollow-dependent species that use hollows in dead trees [50,51] may therefore have an increased risk of heat related impacts if sufficient densities of thermally suitable hollows are not available. Hence, future conservation programs should aim to retain existing live hollow-bearing trees in agricultural landscapes and ensure that there is recruitment of trees via replanting schemes to offset tree loss.

More generally, future conservation programs incorporating the creation of CHs in dead trees should consider how tree size and hollow placement on the tree may influence hollow temperatures. In our study, we found that wall thickness significantly influenced temperatures within CHs, such that thicker-walled hollows had lower maximum temperatures, and lower thermal variation than thinner-walled hollows (Figure 4b,d). Projects in which CHs are carved into dead trees could therefore provide more thermally suitable hollows for wildlife by selecting large trees, and by placing hollows lower down on the trunks of trees where trunk diameter (and thus, wall thickness) is greater. Additional research on other variables that may influence temperatures in CHs in dead trees (e.g., canopy cover, solar radiation, and orientation) would be worth exploring.

Although chainsaw-carved hollows are used by a diversity of wildlife [22,24], and can provide suitable thermal conditions for wildlife [15,26] they also have problems. For example, CHs can only be installed by skilled arborists, and as such, they are expensive to install, and cannot be placed on small trees due to the risks of tree failure. For this reason, it may be necessary to incorporate a diversity of hollow types into conservation programs aimed at restoring landscapes for hollow-dependent wildlife. An additional technique for constructing hollows is to use drills to mechanically create entrances in live trees with internal decay or hidden voids which have not developed entrances. A recent study showed that such hollows were rapidly used by wildlife, and temperatures inside the hollows fluctuated by 4 °C in summer whereas ambient temperatures fluctuated by 21 °C [52]. Hollows created with drills and auger bits may therefore be useful for creating artificial hollows, particularly if the aim is to create small hollows for smaller hollow-dependent animals such as microbats. However, further research into the hollow requirements of Australia’s insectivorous bats is required and artificial hollows with small entrances may require more regular monitoring and maintenance to prevent wound wood growth from closing the entrances. While we now know more about the thermal properties of artificial hollows, more research is needed to establish whether threatened species, which are often the target of habitat restoration studies, will use such structures, and if so, whether the addition of artificial structures can help to offset declines.

## 5. Conclusions

Our results show that the thermal profiles of artificial hollows carved into dead trees were similar to those of natural hollows. However, during the 2019 heatwave, only 3 of 30 CHs carved into dead trees provided suitable thermal regimes for hollow-dependent nocturnal mammals that shelter within hollows during the daytime. In agricultural landscapes, retention of large live trees will be crucial for conserving native wildlife, as live trees typically contain multiple hollows, some of which are likely to be well-buffered against thermal extremes during heatwaves [26]. Future studies aimed at reversing population declines of hollow-dependent wildlife should incorporate revegetation programs in conjunction with the provision of a wide diversity of artificial hollow types, some of which should be installed in live trees where possible.

## Data Availability

Data supporting reported results can be found online in the Supplementary Materials.

## Supplementary Materials

The following supporting information can be downloaded at: https://www.mdpi.com/article/10.3390/biology12030361/s1, File S1: raw data used in statistical analyses.
